# Interaction Analysis between *HLA-DRB1* Shared Epitope Alleles and MHC Class II Transactivator *CIITA* Gene with Regard to Risk of Rheumatoid Arthritis

**DOI:** 10.1371/journal.pone.0032861

**Published:** 2012-03-26

**Authors:** Marcus Ronninger, Maria Seddighzadeh, Morten Christoph Eike, Darren Plant, Nina A. Daha, Beate Skinningsrud, Jane Worthington, Tore K. Kvien, Rene E. M. Toes, Benedicte A. Lie, Lars Alfredsson, Leonid Padyukov

**Affiliations:** 1 Rheumatology Unit, Department of Medicine, Karolinska Institutet and Karolinska University Hospital, Stockholm, Sweden; 2 Department of Medical Genetics, University of Oslo and Oslo University Hospital, Oslo, Norway; 3 1arc-Epidemiology Unit, University of Manchester, Manchester, United Kingdom; 4 Department of Rheumatology, Leiden University Medical Center, Leiden, The Netherlands; 5 Department of Rheumatology, Diakonhjemmet Hospital, Oslo, Norway; 6 Institute of Environmental Medicine, Karolinska Institutet, Stockholm, Sweden; University of Crete, Greece

## Abstract

*HLA-DRB1* shared epitope (SE) alleles are the strongest genetic determinants for autoantibody positive rheumatoid arthritis (RA). One of the key regulators in expression of HLA class II receptors is MHC class II transactivator (*CIITA*). A variant of the *CIITA* gene has been found to associate with inflammatory diseases.

We wanted to explore whether the risk variant rs3087456 in the *CIITA* gene interacts with the *HLA-DRB1* SE alleles regarding the risk of developing RA. We tested this hypothesis in a case-control study with 11767 individuals from four European Caucasian populations (6649 RA cases and 5118 controls).

We found no significant additive interaction for risk alleles among Swedish Caucasians with RA (n = 3869, attributable proportion due to interaction (AP) = 0.2, 95%CI: −0.2–0.5) or when stratifying for anti-citrullinated protein antibodies (ACPA) presence (ACPA positive disease: n = 2945, AP = 0.3, 95%CI: −0.05–0.6, ACPA negative: n = 2268, AP = −0.2, 95%CI: −1.0–0.6). We further found no significant interaction between the main subgroups of SE alleles (DRB1*01, DRB1*04 or DRB1*10) and *CIITA*. Similar analysis of three independent RA cohorts from British, Dutch and Norwegian populations also indicated an absence of significant interaction between genetic variants in *CIITA* and SE alleles with regard to RA risk.

Our data suggest that risk from the *CIITA* locus is independent of the major risk for RA from *HLA-DRB1* SE alleles, given that no significant interaction between rs3087456 and SE alleles was observed. Since a biological link between products of these genes is evident, the genetic contribution from *CIITA* and class II antigens in the autoimmune process may involve additional unidentified factors.

## Introduction

Rheumatoid arthritis (RA) is a relatively common disease of poorly understood aetiology that affects approximately 1% of the world's population. Even though the pathophysiology of the disease is well studied only a limited number of risk factors with low to moderate effect has been described [1]. Even the strongest genetic risk factor for RA, the variants in the *HLA-DRB1* gene suggested by the shared epitope (SE) hypothesis [2], confer only a moderate risk increase for RA, with an odds ratio (OR) of 4–6 in European Caucasians with regard to anti-citrullinated protein antibodies (ACPA) positive RA [3], [4]. Also, these variations are quite common in the normal population and their predictive value is very low. Therefore, the major fraction of RA risk remains unexplained by existing information and interaction between known risk factors may account for putative “missing” risk factors. Indeed, some risk factors for RA has been shown to moderate the risk for disease in the context of the SE [3], [5], [6] suggesting that interactions with SE may play an important role in the development of RA [7].

We have earlier reported on a variant of the MHC class II transactivator (*CIITA*) gene, the −168A/G promoter SNP (rs3087456), which associates with inflammatory diseases and also the expression of *CIITA* and downstream HLA expression [8]. This association was not consistently replicated in different populations [9], [10]. However, other variants in the CIITA locus than rs3087456 have been reported in association with autoimmune disease [11], [12], [13] warranting further exploration of this locus in the context of RA.

Also, due to involvement of HLA class II in RA, the study of *CIITA* may reveal more detailed mechanisms of disease development, since the protein is known to be a key regulator of MHC class II expression and therefore may be involved in development of RA in combination with SE alleles [14], [15]. A complete lack of expression of *CIITA* leads to the bare lymphocyte syndrome with a complete abolishment of classical MHC class II gene expression [16]. This is unlikely to be relevant to RA, but less severe changes in efficiency of CIITA expression might be important for autoimmunity development and statistical evaluation of genetic interaction of CIITA and shared epitope alleles may reveal “missing” risk factors.

With this as a background, we set out to define a possible gene-gene interaction between *HLA-DRB1* and the *CIITA* locus in development of RA with a study population of 11767 individuals from four European Caucasian cohorts (6649 RA cases and 5118 controls).

## Results

First, we tested the hypothesis of an interactive effect between risk alleles of *HLA-DRB1* SE and rs3087456 for developing of RA in the Swedish cohort (Cohort I, Table S1). Interaction was estimated between SE positivity and the risk allele G of rs3087456 in a homozygous state (GG) [8]. The analysis demonstrated no significant evidence of interaction in this model (attributable proportion (AP) = 0.2, 95% CI: −0.2–0.5). Since SE is primarily a risk factor for ACPA positive disease we stratified data according to ACPA status of RA cases. Still, no significant evidence for interaction was found, although a tendency was apparent (AP = 0.3, 95%CI: −0.05–0.6 for ACPA positive status in RA cases, Table 1). Similarly, no significant interaction in additive and multiplicative models was found in the British and the Dutch cohorts. In the Norwegian cohort, however, a significant interaction was detected, both in the total material and in the ACPA positive RA cases (AP = 0.4, 95%CI: 0.03–0.0.7 for RA in total and AP = 0.4, 95%CI: 0.05–0.7 for ACPA positive status in RA cases, Table 1).

**Table 1:** Risk of developing RA for combinations of the *HLA-DRB1* SE and rs3087456 alleles in Swedish.

Sweden. all		Cases/Controls	OR	95% C.I.	AP (95% C.I.)	Add.	Mult.
*CIITA 168GG*	SE				0.2(−0.2–0.5)	P = 0.4	P = 0.9
No	None	571/582	1.0	…			
No	Any	1655/630	2.7	2.3–3.1			
Yes	None	41/30	1.4	0.9–2.3			
Yes	Any	117/33	3.6	2.4–5.4			
Sweden. ACPA+							
*CIITA 168GG*	SE				0.3(−0.05–0.6)	P = 0.1	P = 0.4
No	None	222/582	1.0	…			
No	Any	1205/630	5.0	4.2–6.0			
Yes	None	11/30	1.0	0.5–2.0			
Yes	Any	86/33	6.8	4.4–10.5			
Norway. all							
*CIITA 168GG*	SE				0.4(0.03–0.7)	P = 0.03	P = 0.4
No	None	186/682	1.0	…			
No	Any	533/751	3.8	2.1–3.2			
Yes	None	14/44	0.9	0.6–2.2			
Yes	Any	50/43	3.1	2.7–6.6			
Norway. ACPA+							
*CIITA 168GG*	SE				0.4(0.05–0.7)	P = 0.02	P = 0.7
No	None	56/682	1.0	…			
No	Any	363/751	6.2	4.4–7.9			
Yes	None	5/44	0.7	0.5–3.6			
Yes	Any	35/43	5.5	5.9–16.7			
UK. all							
*CIITA 168GG*	SE				−0.2(−0.7–0.3)	P = 0.4	P = 0.7
No	None	429/638	1.0	…			
No	Any	1354/529	3.0	3.2–4.5			
Yes	None	36/57	0.9	0.6–1.5			
Yes	Any	97/46	2.1	2.2–4.5			
UK. ACPA+							
*CIITA 168GG*	SE				−0.1(−0.5–0.3)	P = 0.7	P = 0.6
No	None	198/638	1.0	…			
No	Any	1023/529	5.9	5.1–7.5			
Yes	None	13/57	1.0	0.4–1.4			
Yes	Any	79/46	4.3	3.7–8.2			
The Netherlands. all							
*CIITA 168GG*	SE				−0.3(−1.4–0.8)	P = 0.6	P = 0.8
No	None	136/146	1.0	…			
No	Any	9/11	2.6	2.2–4.1			
Yes	None	321/116	1.2	0.4–2.2			
Yes	Any	20/10	4.3	1.0–4.8			
The Netherlands. ACPA+							
*CIITA 168GG*	SE				−0.4(−1.7–1.0)	P = 0.6	P = 0.8
No	None	27/146	1.0	…			
No	Any	2/11	5.9	3.7–9.6			
Yes	None	127/116	1.4	0.2–4.7			
Yes	Any	8/10	9.9	1.6–12.0			

British. Dutch and Norwegian cohorts.

Results for additive (add.) and multiplicative (mult.) interaction is displayed as significance (P value) of deviation from expected risk given no interaction. AP = attributable proportion; SE = shared epitope; OR = odds ratio; ACPA+ = anti citrullinated protein antibody positive RA patients; CI = confidence interval.

To investigate the interaction between *HLA-DRB1* SE alleles and rs3087456 in depth, we used a more detailed description of the *HLA-DRB1* SE alleles by introducing the allelic groups DRB1*01, DRB1*04 and DRB1*10 as separate risk factors. These analyses did not reveal an SE subgroup allele specific interaction with SNP rs3087456 (Table 2).

**Table 2:** Summary data of the interaction analysis for *HLA-DRB1* SE allelic groups and SNP rs3087456 for the Swedish cohort.

Group	rs3087456 and:	AP	CI 95 low	CI 95 high	P value
All	SE (yes/no)	0.2	−0.2	0.5	0.5
	DRB1*01	−0.3	−1.2	0.6	0.5
	DRB1*04	0.2	−0.2	0.6	0.3
	DRB1*10	0.05	−1.5	1.6	0.9
ACPA+	SE (yes/no)	0.3	−0.05	0.6	0.1
	DRB1*01	−0.2	−1.1	0.7	0.7
	DRB1*04	0.3	−0.1	0.6	0.2
	DRB1*10	−0.1	−2.0	1.8	0.9

Additive interaction is presented as attributable proportion (AP) with 95% confidence interval (CI). For additional analysis see Table S5. SE = shared epitope; ACPA+ = anti citrullinated protein antibody positive RA patients.

Since other variants in the *CIITA* locus have been reported in association with autoimmune disease we genotyped an additional 22 SNPs across the *CIITA* locus for the Swedish cohort (Chr16: 10842650–10931606, details for RA association tests of these SNPs can be found in Tables S2 and S3). Of these 22 SNPs, including rs3087456, only rs8048002 was significantly associated with RA after correction for multiple comparisons (ACPA negative patients, adjusted for 44 test: p = 0.013, data submitted elsewhere, Eike et al. [17]). Rs3087456 was significantly associated with RA before adjusting for multiple comparisons. To exhaust the possibility of an interaction between *CIITA* variants and SE, we screened all *CIITA* SNPs for interaction in the Swedish cohort, using the additive model and two alternative models of dominance: with the minor and major allele of each SNP. In these analyses the SNP rs4781019 showed significant interaction with SE for the ACPA positive subgroup (Table 3, see Table S4). However, the statistical significance did not hold after correction for multiple testing (nominal p = 0.02 for RA in total, nominal p = 0.03 for ACPA positive RA, significance threshold for 44 tests is p = 0.0011, Bonferroni correction). In addition the SE interaction with this SNP could not be confirmed in the independent Norwegian cohort (Table 3.)

**Table 3:** Summary data for interaction analysis between *CIITA* rs4781019 and *HLA-DRB1* SE.

		Dominant model		Recessive model	
Cohort	Group	AP	P value	AP	P value
Swedish	All	0.3	0.02	0.1	0.5
	ACPA+	0.3	0.03	0.1	0.4
Norwegian	All	0.04	0.7	0.03	0.8
	ACPA+	−0.01	0.9	0.1	0.4

The table presents the best result after analysis of interaction between the *CIITA* locus and *HLA-DRB1*. Dominant and recessive (for the risk allele) genetic models were tested for each SNP, see Table S4 for complete results. AP = attributable proportion; SE = shared epitope; ACPA+ = anti citrullinated protein antibody positive RA patients.

## Discussion

To assess the combinatorial risk of *CIITA* and *HLA-DRB1* we have investigated the interaction between *HLA-DRB1* SE alleles and the *CIITA* −168A/G polymorphism rs3087456, which was previously found to be associated with RA [8]. It may be that the risk for disease is only detectable in certain combinations and also in certain population. This may be the underlying reason why rs3087456 has been shown as a genetic risk factor for immunological disease in some cohorts [8], [18], [19], but has not been consistently replicated in other cohorts [20], [21], [22]. This was why we set out to define a more specific role of this polymorphism through genetic interaction with *HLA-DRB1* SE alleles. However, a straightforward interaction model of SE and the rs3087456 G allele did not reveal significant interaction with regard to RA. In addition, we performed a detailed analysis with the specific SE alleles DRB1*01, DRB1*04, DRB1*10 for a more strict allelic interaction. From this we could conclude that the interaction trend stayed with DRB1*04 but it did not reach statistical significance. We observed that the models with DRB1*01 and DRB1*10 showed negative interaction, which led us to remove individuals with these genotypes from the dataset for a more fair measure of effect from DRB1*04. This analysis resulted in a significant interaction, but was not replicated in the Norwegian cohort (Table S5). A reason for this could be the reduction in size of the dataset and decreased statistical power.

Although the involvement of SNP rs3087456 was the main focus of our study, we also addressed genetic variability in this locus on a broader scale by scrutinizing the *CIITA* locus for other putative risk markers in the Swedish RA cohort. In a recent article addressing the influence of *CIITA* and *HLA-DRB1* in multiple sclerosis [11], a complex risk relationship between these loci is presented. The polymorphism rs4774 is described as the major risk variant in the *CIITA* locus instead of rs3087456 and with an increased risk in individuals carrying the DRB1*1501 allele. Rs4774 is also reported to be associated with the production of donor-specific HLA antibodies in renal allograft recipients [23]. Indeed, we found some evidence for interaction with another polymorphism than rs3087456 (rs4781019), but this could not be replicated in the independent Norwegian cohort.

In general, it is difficult to estimate the statistical power for measuring low effects of interaction. Also, except for the Swedish cohort, no particular measures were made to match controls with the RA patients, which may reduce the power of our study and to increase genetic heterogeneity of the study. Therefore, to conclude that an interaction is completely absent between *CIITA* and *HLA-DRB1* SE alleles in development of RA is not possible. However, absence of convincing results in four large, independent cohorts makes it highly unlikely that any strong interaction is present or a sizeable subgroup of the disease could be explained by this interaction.

Our study is not directly comparable to association studies of *CIITA* in RA, since the major aim was to discover a hypothetical interaction between *CIITA* and *HLA-DRB1* SE. Even so, this and previous studies indicates that *CIITA* plays an ambiguous role for RA where association signals are difficult to replicate. In a recent article by Eike et al. (unpublished) [17], an updated meta-analysis supports the association of *CIITA* with RA, in particular in the Scandinavian populations. Also, it seems likely that *CIITA* is involved in other autoimmune disease, with multiple sclerosis being the most pronounced [11], [18], [24], [25]. According to our data, the previously found association of CIITA variations with RA that could not be replicated in different Caucasian populations is not due to a putative interaction between CIITA and SE alleles. Thus, the missing genetic risk factors for RA remain to be discovered.

To conclude, we did not observe any significant interaction between rs3087456 or 22 other SNPs in the *CIITA* locus and *HLA-DRB1* SE alleles with regard to risk of RA.

## Materials and Methods

### Description of cohorts

Interaction between HLA-DRB1 SE alleles and rs3087456 in *CIITA* was primarily investigated in a cohort consisting of 2520 incidence RA cases and 1349 matched controls from Swedish EIRA study, which is described elsewhere [3], [26]. The analysis was repeated in three other cohorts: from UK (1916 cases and 1270 controls), the Netherlands (1260 cases and 346 controls) and Norway (953 cases and 2153 controls) with overall 11767 individuals in the study (6649 RA cases and 5118 controls). All RA patients met the American College for Rheumatology 1987 (ACR-87) revised criteria for RA [27]. For the Swedish cohort, patients were recruited to the study by practioners not responsible for the study, who after informing registered a verbal consent in the patients journal. If consent was given, an extensive questionere was filled in by the patient. Controls were invited by letter and were also asked to fill in and send back an extensive questionere and to visit the closest primary care for leaving samples. This active participation was the foundation for informed consent. All subjects can at any moment withdraw from the study. This procedure is in line with the ethical permit and regulation in Sweden. All individuals in the Dutch, Brittish and Norwegian cohort gave their written informed consent to participate. The local regional ethical review boards approved this study (Regionala etikprövningsnämnden i Stockholm, Sweden; The Leiden institutional review board, Commissie Medische Ethiek, Netherlands; The North-West Multi-Centre Research Ethics Committee and University of Manchester Committee on the Ethics of Research on Human Beings, United Kingdom; Regionale komiteer for medisinsk og helsefaglig forskningsetikk (REK) sør-øst, Oslo, Norway).

### Swedish cohort

Genotyping for Swedish cohort was performed by TaqMan predesigned genotyping assay (*CIITA*) (Applied Biosystems, Foster City, California) and by SSP-PCR (Olerup SSP, Saltsjöbaden, Sweden). The ACPA status was previously identified for all RA patients by Immunoscan RA (Mark 2, cut-off for positivity ≥25 U/ml) enzyme-linked immunosorbent assay (Euro-Diagnostica, Malmö, Sweden).

In the EIRA study, only a minor number of controls were detected to be ACPA positive (1.8%, n = 24), while controls from other cohorts were not tested. When stratifying by ACPA status, controls were always considered as a whole group and not divided in strata.

### Dutch cohort

Patient characteristics have been described previously [28]. The healthy controls were randomely selected by the immunogenetics and Transplantation Immunology section of the Leiden University Medical Center.

Genotyping was performed as described for the Swedish cohort. ACPA status was determined by Immunoscan RA (Mark 2, cut-off for positivity ≥25 U/ml) enzyme-linked immunosorbent assay (Euro-Diagnostica, Malmö, Sweden). HLA typing has been described elsewhere [29].

### British cohort

All subjects were white Caucasians and all patients satisfied ACR-87 criteria modified for genetic studies [30].

Genotyping for the UK cohort was performed using the Sequenom MassARRAY iPLEX system in accordance with the manufacturer's instructions (www.Sequenom.com). ACPA was tested using the Axis-Shield DIASTAT kit according to the manufacturer's instructions (positivity: concentration >5 U/ml). The presence of *HLA-DRB1* SE copy number (0, 1 or 2 copies) was detected using a semi-automated reverse hybridization method (Dynal Biotech, Wirral, UK).

### Norwegian cohort

Norwegian RA patients were from the Oslo RA Registry (ORAR) and the European Research on Incapacitating Disease and Social Support (EURIDISS) cohorts [31], [32]. Healthy Norwegian control samples were collected from the Norwegian Bone Marrow Donor Registry (NBMDR), Oslo University Hospital, Rikshospitalet (Controls-1, n = 1121) and blood donors recruited at Oslo University Hospital, Ullevål (Controls-2, n = 1032). An ELISA kit assay (INOVA Diagnostics, San Diego, California, USA) was used to measure ACPA concentrations in the RA samples, with a positivity cut-off defined as levels >25 U/ml. Genotyping of *CIITA* SNPs in the Norwegian cohort was performed with TaqMan predesigned assays. Genotyping for *HLA-DRB1* in the Norwegian RA patients and controls from NBMDR was done by sequence-based genotyping [33], whereas blood donors were genotyped by PCR-based sequence-specific oligonucleotide probe system [34].

### Statistical analyses

Additive interaction was defined by departure from additivity of effects originally described by Rothman [35] and was estimated by calculating the attributable proportion due to interaction (AP) [36]. For each individual, variables were defined for having none, either or both risk factors. The statistical tool R was used for logistic regression and estimating ORs for variables and AP was calculated with 95% confidence intervals (version 2.9.0, http://www.r-project.org/). This procedure was facilitated by scripts developed by Kallberg et al., 2006 [37]. Interaction was calculated between SNP rs3087456 (homozygous for G allele) and *HLA-DRB1* SE alleles defined as any of DRB1*01, DRB1*04 and DRB1*10. For the other polymorphisms in the *CIITA* locus both dominant and recessive models for the risk allele were used. For calculating multiplicative interaction and performing allelic association analysis we used the software PLINK (version 1.06, http://pngu.mgh.harvard.edu/purcell/plink/).

We used Bonferroni correction for multiple testing when appropriate.

## Supporting Information

Table S1 **Description of the cohorts included in the study.**
(DOC)

Table S2 **Association analyses of the CIITA locus with risk of RA in the Swedish cohort.** Pos refers to the genomic position in chromosome 16. MAF is short for minor allele frequency. Plink was used for statistical analysis (http://pngu.mgh.harvard.edu/purcell/plink/). P-values are unadjusted.
(DOC)

Table S3 **Additional data from association analysis.** P values were calculated with trend test in Plink (http://pngu.mgh.harvard.edu/purcell/plink/). P values are unadjusted.
(DOC)

Table S4 **A summary of interaction analysis for** ***HLA-DRB1*** **SE with the CIITA locus for the Swedish cohort.** * For recessive models the complementary to the recessive risk allele is used for calcualtion due to a low allele frequency. AP = attributable proportion; SE = shared epitope; ACPA = anit citrullinated protein antibodies. Positions with missing data (−) were not possible to calculate.
(DOC)

Table S5 **Additive interaction between rs3087456 and** ***HLA-DRB1*** **SE subgroups.** # Exclusion of individuals with DBR1*01 or DRB1*10 alleles. Interaction between rs3087456 and DBR1*10 could not be calculated for the Norwegian cohort. AP = attributable proportion; SE = shared epitope; ACPA = anit citrullinated protein antibodies.
(DOC)
