# Supplementary material for: Interaction Analysis between HLA-DRB1 Shared Epitope Alleles and MHC Class II Transactivator CIITA Gene with Regard to Risk of Rheumatoid Arthritis
Source: PLoS One. 2012 Mar 26;7(3):e32861. doi: 10.1371/journal.pone.0032861 (PMC3312880; doi:10.1371/journal.pone.0032861)
Supplement: Table S1 — Description of the cohorts included in the study. (DOC) [file pone.0032861.s001.doc]

**Table S1**. Description of the cohorts included in the study

|  | No | Females. % | Males. % | ACPA pos.% | ACPA neg. % |
| --- | --- | --- | --- | --- | --- |
| Cohort I. Sweden |  |  |  |  |  |
| Patients | 2520 | 71.4 (1800) | 28.6 (720) | 63.5 (1596) | 36.5 (919) |
| Controls | 1349 | 73.5 (991) | 26.5 (358) | 1.8 (24) | 98.2 (1325) |
| Cohort II. Leiden |  |  |  |  |  |
| Patients | 1260 | 64.8 (774) | 35.2 (420) | 57.8 (394) | 42.2 (288) |
| Controls | 346 | 46.4 (130) | 53.6 (150) | - | - |
| Cohort III. UK |  |  |  |  |  |
| Patients | 1916 | 71.7 (1374) | 28.3 (542) | 68.5 (1313) | 31.5 (603) |
| Controls | 1270 | 59.5 (756) | 40.5 (514) | - | - |
| Cohort IV. Norway |  |  |  |  |  |
| Patients | 953 | 77.3 (737) | 22.7 (216) | 61.6 (550) | 38.4 (343) |
| Controls-I | 1121 | 55.0 (617) | 45.0 (504) | - | - |
| Controls-II | 1032 | - | - | - | - |
| Total | 11767 |  |  |  |  |
| Patients | 6649 | 71.2 (4685) | 28.8 (1898) |  |  |
| Controls | 5118 | 62.0 (2494) | 38.0 (1526) |  |  |
