# Supplementary material for: Interaction Analysis between HLA-DRB1 Shared Epitope Alleles and MHC Class II Transactivator CIITA Gene with Regard to Risk of Rheumatoid Arthritis
Source: PLoS One. 2012 Mar 26;7(3):e32861. doi: 10.1371/journal.pone.0032861 (PMC3312880; doi:10.1371/journal.pone.0032861)
Supplement: Table S2 — Association analyses of the CIITA locus with risk of RA in the Swedish cohort. Pos refers to the genomic position in chromosome 16. MAF is short for minor allele frequency. Plink was used for statistical analysis (http://pngu.mgh.harvard.edu/purcell/plink/). P-values are unadjusted. (DOC) [file pone.0032861.s002.doc]

**Table S2**. Association analyses of the CIITA locus with risk of RA in the Swedish cohort

| Allelic association test | | All individuals | | | |  | ACPA positive | | | |  | ACPA negative | | | |
| --- | --- | --- | --- | --- | --- | --- | --- | --- | --- | --- | --- | --- | --- | --- | --- |
| SNP | Pos | MAF Patients | MAF Controls | OR (95% CI) | P |  | MAF Patients | MAF Controls | OR (95% CI) | P |  | MAF Patients | MAF Controls | OR (95% CI) | P |
| rs11074930 | 10842650 | 0.489 | 0.498 | 0.97 (0.87-1.07) | 0.51 |  | 0.482 | 0.498 | 0.94 (0.84-1.05) | 0.28 |  | 0.501 | 0.498 | 1.01 (0.89-1.16) | 0.85 |
| rs10431908 | 10851548 | 0.226 | 0.201 | 1.16 (1.02-1.32) | 0.02 |  | 0.228 | 0.201 | 1.18 (1.02-1.36) | 0.02 |  | 0.222 | 0.201 | 1.13 (0.97-1.33) | 0.12 |
| rs8052975 | 10856764 | 0.254 | 0.227 | 1.16 (1.03-1.32) | 0.02 |  | 0.258 | 0.227 | 1.19 (1.04-1.36) | 0.01 |  | 0.247 | 0.227 | 1.12 (0.96-1.31) | 0.15 |
| rs4781003 | 10859668 | 0.166 | 0.151 | 1.12 (0.97-1.29) | 0.13 |  | 0.167 | 0.151 | 1.13 (0.96-1.32) | 0.14 |  | 0.164 | 0.151 | 1.11 (0.92-1.32) | 0.27 |
| rs7501308 | 10862957 | 0.256 | 0.233 | 1.13 (1.00-1.28) | 0.05 |  | 0.262 | 0.233 | 1.17 (1.02-1.34) | 0.02 |  | 0.246 | 0.233 | 1.08 (0.92-1.26) | 0.36 |
| rs4781009 | 10865178 | 0.255 | 0.228 | 1.16 (1.02-1.32) | 0.03 |  | 0.254 | 0.228 | 1.16 (1.00-1.33) | 0.05 |  | 0.255 | 0.228 | 1.16 (0.99-1.37) | 0.07 |
| rs6498114 | 10871619 | 0.233 | 0.221 | 1.07 (0.94-1.22) | 0.29 |  | 0.239 | 0.221 | 1.11 (0.96-1.28) | 0.16 |  | 0.224 | 0.221 | 1.02 (0.86-1.21) | 0.80 |
| rs6416647 | 10873098 | 0.291 | 0.254 | 1.21 (1.06-1.36) | 0.003 |  | 0.290 | 0.254 | 1.20 (1.05-1.38) | 0.009 |  | 0.293 | 0.254 | 1.21 (1.04-1.42) | 0.02 |
| rs11074932 | 10875837 | 0.283 | 0.253 | 1.17 (1.03-1.32) | 0.01 |  | 0.283 | 0.253 | 1.17 (1.02-1.34) | 0.03 |  | 0.284 | 0.253 | 1.17 (1.00-1.37) | 0.05 |
| rs6498116 | 10876783 | 0.219 | 0.192 | 1.18 (1.03-1.36) | 0.02 |  | 0.218 | 0.192 | 1.17 (1.01-1.36) | 0.04 |  | 0.223 | 0.192 | 1.21 (1.02-1.43) | 0.03 |
| rs3087456 | 10878403 | 0.247 | 0.224 | 1.14 (1.02-1.27) | 0.02 |  | 0.250 | 0.224 | 1.15 (1.02-1.30) | 0.02 |  | 0.243 | 0.224 | 1.11 (0.97-1.28) | 0.14 |
| rs4781011 | 10882812 | 0.239 | 0.207 | 1.20 (1.04-1.39) | 0.01 |  | 0.240 | 0.207 | 1.22 (1.04-1.43) | 0.02 |  | 0.236 | 0.207 | 1.19 (0.99-1.42) | 0.06 |
| rs8048002 | 10899489 | 0.070 | 0.052 | 1.37 (1.12-1.67) | 0.003 |  | 0.065 | 0.052 | 1.28 (1.02-1.59) | 0.03 |  | 0.078 | 0.052 | 1.53 (1.20-1.95) | 0.0006 |
| rs6498124 | 10903351 | 0.445 | 0.457 | 0.95 (0.84-1.07) | 0.41 |  | 0.435 | 0.457 | 0.91 (0.80-1.04) | 0.18 |  | 0.461 | 0.457 | 1.01 (0.87-1.18) | 0.87 |
| rs11647384 | 10904790 | 0.396 | 0.404 | 0.97 (0.86-1.10) | 0.61 |  | 0.392 | 0.404 | 0.95 (0.83-1.09) | 0.50 |  | 0.403 | 0.404 | 1.00 (0.86-1.16) | 0.96 |
| rs4774 | 10908349 | 0.294 | 0.280 | 1.07 (0.91-1.26) | 0.41 |  | 0.295 | 0.280 | 1.08 (0.91-1.28) | 0.40 |  | 0.293 | 0.280 | 1.06 (0.88-1.28) | 0.53 |
| rs4781019 | 10911651 | 0.449 | 0.473 | 0.91 (0.81-1.03) | 0.13 |  | 0.441 | 0.473 | 0.88 (0.77-1.01) | 0.06 |  | 0.463 | 0.473 | 0.96 (0.83-1.12) | 0.64 |
| rs11074938 | 10914044 | 0.373 | 0.344 | 1.13 (1.00-1.28) | 0.05 |  | 0.369 | 0.344 | 1.11 (0.97-1.28) | 0.14 |  | 0.381 | 0.344 | 1.17 (1.00-1.37) | 0.05 |
| rs8056269 | 10920068 | 0.429 | 0.416 | 1.05 (0.93-1.19) | 0.40 |  | 0.436 | 0.416 | 1.09 (0.95-1.24) | 0.23 |  | 0.416 | 0.416 | 1.00 (0.86-1.17) | 0.98 |
| rs1139564 | 10926123 | 0.161 | 0.150 | 1.08 (0.92-1.28) | 0.35 |  | 0.165 | 0.150 | 1.11 (0.93-1.34) | 0.25 |  | 0.154 | 0.150 | 1.03 (0.84-1.27) | 0.79 |
| rs8052709 | 10927756 | 0.208 | 0.210 | 0.99 (0.85-1.15) | 0.88 |  | 0.197 | 0.210 | 0.92 (0.78-1.09) | 0.35 |  | 0.224 | 0.210 | 1.09 (0.91-1.31) | 0.36 |
| rs4072865 | 10931606 | 0.487 | 0.467 | 1.08 (0.96-1.22) | 0.19 |  | 0.482 | 0.467 | 1.06 (0.93-1.22) | 0.36 |  | 0.493 | 0.467 | 1.11 (0.96-1.29) | 0.17 |

Pos refers to the genomic position in chromosome 16. MAF is short for minor allele frequency. Plink was used for statistical analysis (<http://pngu.mgh.harvard.edu/purcell/plink/>). P-values are unadjusted
