# Supplementary material for: Interaction Analysis between HLA-DRB1 Shared Epitope Alleles and MHC Class II Transactivator CIITA Gene with Regard to Risk of Rheumatoid Arthritis
Source: PLoS One. 2012 Mar 26;7(3):e32861. doi: 10.1371/journal.pone.0032861 (PMC3312880; doi:10.1371/journal.pone.0032861)
Supplement: Table S3 — Additional data from association analysis. P values were calculated with trend test in Plink (http://pngu.mgh.harvard.edu/purcell/plink/). P values are unadjusted. (DOC) [file pone.0032861.s003.doc]

**Table S3**. Additional data from association analysis

|  | P-value for trend test | | |  | Numer of individuals in analysis | | |  | HWE test | | |
| --- | --- | --- | --- | --- | --- | --- | --- | --- | --- | --- | --- |
| SNP | All | ACPA pos | ACPA neg |  | All | ACPA pos | ACPA neg |  | Control | Case | All |
| rs11074930 | 0.51 | 0.28 | 0.85 |  | 3106 | 2326 | 1803 |  | 1 | 0.96 | 0.97 |
| rs10431908 | 0.03 | 0.02 | 0.13 |  | 3135 | 2343 | 1829 |  | 0.25 | 0.75 | 0.71 |
| rs8052975 | 0.02 | 0.01 | 0.15 |  | 3144 | 2356 | 1835 |  | 0.29 | 0.56 | 0.88 |
| rs4781003 | 0.14 | 0.14 | 0.27 |  | 3128 | 2341 | 1828 |  | 0.72 | 0.10 | 0.11 |
| rs7501308 | 0.05 | 0.025 | 0.36 |  | 3048 | 2285 | 1774 |  | 0.16 | 0.44 | 0.88 |
| rs4781009 | 0.03 | 0.05 | 0.07 |  | 2706 | 2061 | 1678 |  | 0.33 | 0.70 | 0.79 |
| rs6498114 | 0.28 | 0.16 | 0.80 |  | 2737 | 2089 | 1688 |  | 0.86 | 0.12 | 0.16 |
| rs6416647 | 0.003 | 0.008 | 0.02 |  | 2710 | 2073 | 1669 |  | 1 | 0.68 | 0.74 |
| rs11074932 | 0.02 | 0.03 | 0.05 |  | 2758 | 2099 | 1685 |  | 1 | 0.91 | 0.89 |
| rs6498116 | 0.02 | 0.04 | 0.03 |  | 2727 | 2084 | 1689 |  | 0.69 | 0.32 | 0.56 |
| rs3087456 | 0.03 | 0.02 | 0.15 |  | 3811 | 2909 | 2232 |  | 0.27 | 0.02 | 0.007 |
| rs4781011 | 0.01 | 0.02 | 0.07 |  | 2320 | 1745 | 1433 |  | 0.47 | 0.47 | 0.31 |
| rs8048002 | 0.003 | 0.04 | 0.0007 |  | 3792 | 2900 | 2216 |  | 0.40 | 0.008 | 0.006 |
| rs6498124 | 0.42 | 0.18 | 0.87 |  | 2332 | 1749 | 1426 |  | 0.10 | 0.02 | 0.45 |
| rs11647384 | 0.62 | 0.50 | 0.96 |  | 2331 | 1745 | 1420 |  | 1 | 0.19 | 0.30 |
| rs4774 | 0.41 | 0.40 | 0.53 |  | 1958 | 1381 | 1065 |  | 0.65 | 0.53 | 0.78 |
| rs4781019 | 0.13 | 0.07 | 0.64 |  | 2307 | 1732 | 1409 |  | 0.73 | 0.71 | 0.59 |
| rs11074938 | 0.05 | 0.13 | 0.05 |  | 2305 | 1747 | 1418 |  | 0.60 | 0.54 | 0.41 |
| rs8056269 | 0.41 | 0.24 | 0.98 |  | 2347 | 1766 | 1449 |  | 0.14 | 0.12 | 0.03 |
| rs1139564 | 0.36 | 0.25 | 0.79 |  | 2351 | 1766 | 1445 |  | 0.50 | 0.07 | 0.06 |
| rs8052709 | 0.88 | 0.35 | 0.36 |  | 2316 | 1738 | 1420 |  | 0.92 | 0.43 | 0.61 |
| rs4072865 | 0.19 | 0.36 | 0.17 |  | 2308 | 1722 | 1399 |  | 0.94 | 0.35 | 0.50 |

P values were calculated with trend test in Plink (<http://pngu.mgh.harvard.edu/purcell/plink/>). P values are unadjusted
