# Supplementary material for: Interaction Analysis between HLA-DRB1 Shared Epitope Alleles and MHC Class II Transactivator CIITA Gene with Regard to Risk of Rheumatoid Arthritis
Source: PLoS One. 2012 Mar 26;7(3):e32861. doi: 10.1371/journal.pone.0032861 (PMC3312880; doi:10.1371/journal.pone.0032861)
Supplement: Table S4 — A summary of interaction analysis for HLA-DRB1 SE with the CIITA locus for the Swedish cohort. * For recessive models the complementary to the recessive risk allele is used for calcualtion due to a low allele frequency. AP = attributable proportion; SE = shared epitope; ACPA = anit citrullinated protein antibodies. Positions with missing data (−) were not possible to calculate. (DOC) [file pone.0032861.s004.doc]

**Table S4**. A summary of interaction analysis for *HLA-DRB1* SE with the CIITA locus for the Swedish cohort

| Swedish cohort | |  |  |  |  |  |  |  |  |  |  |  |
| --- | --- | --- | --- | --- | --- | --- | --- | --- | --- | --- | --- | --- |
|  | All individuals | | | | ACPA positive | | | | ACPA negative | | | |
|  | Dominant modell | | Recessive modell | | Dominant modell | | Recessive modell | | Dominant modell | | Recessive modell | |
| snp | AP | P value | AP | P value | AP | P value | AP | P value | AP | P value | AP | P value |
| rs11074930 | -0.18 | 0.25 | 0.10 | 0.42 | 0.12 | 0.32 | 0.08 | 0.51 | -0.33 | 0.20 | -0.34 | 0.23 |
| rs10431908 | 0.03 | 0.77 | -0.15 | 0.66 | 0.11 | 0.28 | -0.12 | 0.71 | -0.19 | 0.37 | -0.24 | 0.67 |
| rs8052975 | 0.05 | 0.63 | -0.34 | 0.32 | 0.12 | 0.25 | -0.19 | 0.52 | -0.15 | 0.46 | -0.82 | 0.27 |
| rs4781003 | 0.03 | 0.82 | -1.04 | 0.24 | 0.05 | 0.68 | -0.59 | 0.35 | -0.05 | 0.81 | -2.59 | 0.28 |
| rs7501308 | 0.01 | 0.91 | -0.42 | 0.24 | 0.07 | 0.50 | -0.29 | 0.35 | -0.17 | 0.42 | -0.87 | 0.27 |
| rs4781009 | 0.00 | 0.97 | -0.51 | 0.21 | 0.07 | 0.56 | -0.27 | 0.41 | -0.18 | 0.39 | -1.32 | 0.19 |
| rs6498114 | -0.05 | 0.69 | -0.60 | 0.19 | 0.08 | 0.45 | -0.26 | 0.46 | -0.47 | 0.07 | -2.21 | 0.16 |
| rs6416647 | -0.01 | 0.92 | -0.37 | 0.29 | 0.11 | 0.30 | 0.03 | 0.89 | -0.36 | 0.11 | -1.70 | 0.10 |
| rs11074932 | 0.02 | 0.90 | -0.30 | 0.36 | 0.11 | 0.31 | 0.03 | 0.89 | -0.25 | 0.23 | -1.36 | 0.13 |
| rs6498116 | -0.05 | 0.72 | 0.13 | 0.65 | 0.06 | 0.58 | -0.07 | 0.60 | -0.36 | 0.15 | -0.41 | 0.55 |
| rs3087456 | 0.07 | 0.47 | 0.15 | 0.44 | 0.10 | 0.28 | 0.27 | 0.09 | -0.02 | 0.92 | -0.19 | 0.65 |
| rs4781011 | 0.07 | 0.60 | -0.01 | 0.99 | 0.11 | 0.34 | 0.16 | 0.56 | -0.06 | 0.77 | -0.33 | 0.59 |
| rs8048002 | 0.14 | 0.35 | - | - | 0.22 | 0.09 | - | - | -0.08 | 0.77 | - | - |
| rs6498124 | -0.11 | 0.54 | 0.27 | 0.01 | 0.03 | 0.86 | -0.03 | 0.86 | -0.30 | 0.34 | 0.11 | 0.57 |
| rs11647384 | -0.39 | 0.10 | -0.39 | 0.10 | -0.32 | 0.16 | -0.32 | 0.16 | -0.55 | 0.17 | 0.03 | 0.89 |
| rs4774 | -0.25 | 0.37 | 0.11 | 0.35 | -0.21 | 0.45 | -0.21 | 0.45 | -0.37 | 0.44 | 0.02 | 0.93 |
| rs34654419 | -0.18 | 0.63 | -0.18 | 0.63 | -0.10 | 0.78 | -0.10 | 0.78 | -0.38 | 0.53 | -0.39 | 0.58 |
| rs4781019 | 0.29 | 0.02 | 0.10 | 0.46 | 0.26 | 0.03 | 0.11 | 0.37 | 0.36 | 0.08 | 0.06 | 0.79 |
| rs11074938 | -0.03 | 0.83 | -0.37 | 0.18 | -0.04 | 0.75 | -0.36 | 0.19 | 0.00 | 0.99 | -0.41 | 0.34 |
| rs8056269 | 0.08 | 0.50 | 0.07 | 0.67 | 0.07 | 0.55 | 0.04 | 0.78 | -0.17 | 0.54 | -0.12 | 0.60 |
| rs1139564 | -0.05 | 0.74 | 0.17 | 0.63 | 0.03 | 0.84 | 0.17 | 0.65 | -0.26 | 0.34 | 0.17 | 0.74 |
| rs8052709 | -0.01 | 0.97 | 0.00 | 0.98 | -0.18 | 0.65 | 0.12 | 0.31 | -0.33 | 0.18 | 0.23 | 0.53 |
| rs4072865 | 0.10 | 0.43 | -0.07 | 0.69 | 0.10 | 0.46 | -0.14 | 0.43 | 0.12 | 0.57 | 0.10 | 0.65 |
|  |  |  |  |  |  |  |  |  |  |  |  |  |
| Norwegian cohort | |  |  |  |  |  |  |  |  |  |  |  |
|  | All individuals | | | | ACPA positive | | | | ACPA negative | | | |
|  | Dominant modell | | Recessive modell | | Dominant modell | | Recessive modell | | Dominant modell | | Recessive modell | |
| snp | AP | P value | AP | P value | AP | P value | AP | P value | AP | P value | AP | P value |
| rs3087456 | -0.07 | 0.58 | 0.35 | 0.03 | -0.06 | 0.66 | 0.37 | 0.02 | 0.10 | 0.67 | 0.16 | 0.71 |
| rs8048002* | 0.13 | 0.50 | -0.14 | 0.56 | 0.31 | 0.02 | -0.43 | 0.09 | -0.60 | 0.24 | - | - |
| rs4781019 | 0.04 | 0.75 | 0.03 | 0.82 | -0.01 | 0.94 | 0.12 | 0.37 | 0.09 | 0.75 | -0.26 | 0.48 |
|  | | | | | | | | | |  |  |  |

* For recessive models the complementary to the recessive risk allele is used for calcualtion due to a low allele frequency.

AP = attributable proportion; SE = shared epitope; ACPA = anit citrullinated protein antibodies. Positions with missing data (-) were not possible to calculate.
