# Supplementary material for: Interaction Analysis between HLA-DRB1 Shared Epitope Alleles and MHC Class II Transactivator CIITA Gene with Regard to Risk of Rheumatoid Arthritis
Source: PLoS One. 2012 Mar 26;7(3):e32861. doi: 10.1371/journal.pone.0032861 (PMC3312880; doi:10.1371/journal.pone.0032861)
Supplement: Table S5 — Additive interaction between rs3087456 and HLA-DRB1 SE subgroups. # Exclusion of individuals with DBR1*01 or DRB1*10 alleles. Interaction between rs3087456 and DBR1*10 could not be calculated for the Norwegian cohort. AP = attributable proportion; SE = shared epitope; ACPA = anit citrullinated protein antibodies. (DOC) [file pone.0032861.s005.doc]

**Tables S5**. Additive interaction between rs3087456 and *HLA-DRB1* SE subgroups.

| **Swedish cohort** | rs3087456 and: | AP | CI 95 low | CI 95 high | P value |
| --- | --- | --- | --- | --- | --- |
| All | *DRB1**04(#) | 0.3 | -0.04 | 0.7 | 0.08 |
| ACPA+ | *DRB1**04(#) | 0.4 | 0.05 | 0.7 | 0.02 |
| **Norwergian cohort** | |  |  |  |  |
| All | *DRB1**01 | 0.2 | -0.4 | 0.8 | 0.5 |
|  | *DRB1**04 | 0.2 | -0.1 | 0.6 | 0.2 |
|  | *DRB1**04(#) | 0.2 | -0.3 | 0.6 | 0.5 |
| ACPA+ | *DRB1**01 | -0.1 | -1.2 | 0.9 | 0.8 |
|  | *DRB1**04 | 0.3 | -0.06 | 0.7 | 0.1 |
|  | *DRB1**04(#) | 0.2 | -0.3 | 0.7 | 0.5 |

# Exclusion of individuals with *DBR1**01 or *DRB1**10 alleles. Interaction between rs3087456 and *DBR1**10 could not be calculated for the Norwegian cohort. AP = attributable proportion; SE = shared epitope; ACPA = anit citrullinated protein antibodies.
